# Cardio-oncology in Latin America and the Caribbean. Current state

**DOI:** 10.3332/ecancer.2025.2014

**Published:** 2025-10-17

**Authors:** Manuel Bazan, Claudia Gutiérrez-Villamil, Amalia Peix, Saurabh Malhotra, Fernando Dettori, Roberto N Agüero, Belén Flores, Claudio Tinoco Mesquita, Enrique Hiplan, Teresa Massardo, Isabel Berrocal, José A Coss, Verónica Gómez, María C Fonseca, Karla Abadí, Adriana Puente, Víctor Rosales, Luis F Chen, Yariela Herrera, Marina Arnal, Aurelio Mendoza, Omar Alonso, Jorge E Aguiar, Carla Cueva, Enrique Estrada, Diana Páez

**Affiliations:** 1 Cardio-Oncology Service, Institute of Oncology and Radiobiology, Havana, Cuba; 2 Nuclear Medicine Service, Fundación Cardioinfantil-La Cardi, Bogotá, Colombia; 3 Institute of Cardiology and Cardiovascular Surgery, Havana, Cuba; 4 Division of Cardiology, Cook County Health, Chicago, IL, USA; 5 Nuclear Medicine, National Atomic Energy Commission, Buenos Aires, Argentina; 6 Bolivian Nuclear Energy Agency (ABEN), Nuclear Medicine Center, El Alto, Bolivia; 7 Antonio Pedro University Hospital, Niteroi, Rio de Janeiro, Brazil; 8 Clinical Nuclear Medicine Service, Base Hospital of Valdivia, Los Ríos, Chile; 9 Universidad de Chile, Hospital Clínico, Santiago, Chile; 10 San Juan de Dios Hospital, Caja Costarricense de Seguro Social, San José, Costa Rica; 11 Rosa Emilia Sanchez de Tavares National Cancer Institute (INCART), Santo Domingo, Dominican Republic; 12 Hospital Oncológico del ISSS, San Salvador, El Salvador; 13 Roosevelt Hospital, Guatemala City, Guatemala; 14 San Felipe General Hospital and Asilo de Inválidos, Tegucigalpa, Honduras; 15 Centro Medico Nacional 20 de Noviembre, ISSTE, CDMX, Mexico; 16 National Autonomous University of Nicaragua (UNAN), Roberto Calderón Gutiérrez Public Hospital, Managua, Nicaragua; 17 National Oncology Institute, Panama City, Panama; 18 Santo Tomás Hospital, Panama City, Panama; 19 La Costa Medical Center, Asuncion, Paraguay; 20 National Cardiovascular Institute INCOR, Lima, Peru; 21 Nuclear Medicine Center, Hospital de Clínicas, Montevideo, Uruguay; 22 Latin America and the Caribbean Division, Department of Technical Cooperation, International Atomic Energy Agency, Vienna, Austria; 23 Nuclear Medicine and Diagnostic Imaging Section, Division of Human Health, Department of Nuclear Sciences and Applications. International Atomic Energy Agency, Vienna, Austria

**Keywords:** cardio-oncology, cardiotoxicity, multimodality imaging

## Abstract

Health problems in the Latin American and Caribbean (LAC) region are mainly associated with noncommunicable diseases, with cardiovascular disease and cancer being the leading causes of death. However, knowledge and training opportunities in cardio-oncology, as well as active cardio-oncology groups, are mainly limited to large academic institutions or isolated private groups. To contribute to the implementation of viable strategies to ensure equitable access to care for all, it is essential to understand the current situation. This publication assesses the epidemiological situation of cancer in LAC and discusses the development of cardio-oncology in the region. It analyses the results of the survey on knowledge and medical action in cardio-oncology carried out among a group of physicians involved in the care of oncology patients and proposes recommendations based on the results obtained.

## Introduction

Health problems in the Latin America and Caribbean (LAC) region are mainly associated with noncommunicable diseases (NCDs) [1, 2].

Increased life expectancy, as well as inter-related factors such as globalisation, urbanisation, the increase in diabetes mellitus, inadequate diet, obesity and physical inactivity, contribute to cardiovascular diseases (CVD) being the leading cause of death in the world [3].

**Figure 1.**
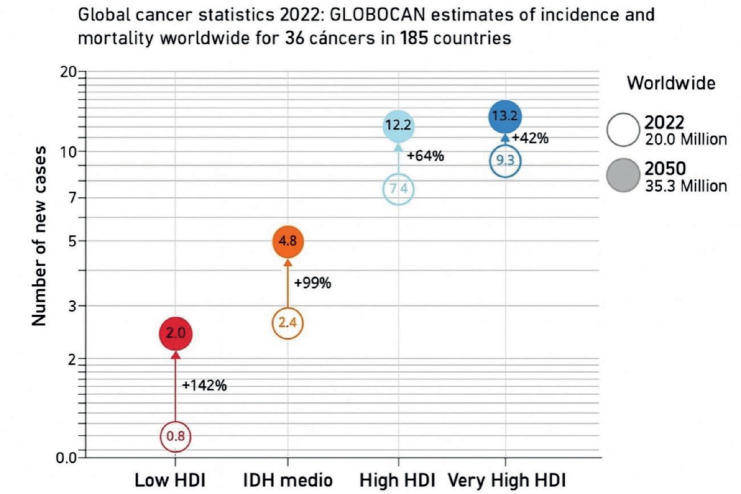
Comparison of new reported cases of cancer between the different groups of countries according to the Human Development Index and the estimated 2050.

On the other hand, globally cancer is the second leading cause of mortality, causing almost 10 million deaths in 2022 (Figure 1), which represents approximately double the figure in 1990 [4]. It is estimated that 40% of cancers can be prevented through healthy lifestyles and that one third of cancers can be diagnosed in their early stages of development through early detection programs [3].

Since the 1950s, the concept of a cure for cancer emerged, which became a reality in the 1990s with the appearance of specific treatments [4]. Since then, there has been talk of a reduction in cancer mortality, but this has been accompanied by increased cardiovascular morbidity and mortality in cancer survivors due to cardiotoxicity [5].

CVD and cancer combined accounted for 65% of all premature deaths [6].

**Figure 2.**
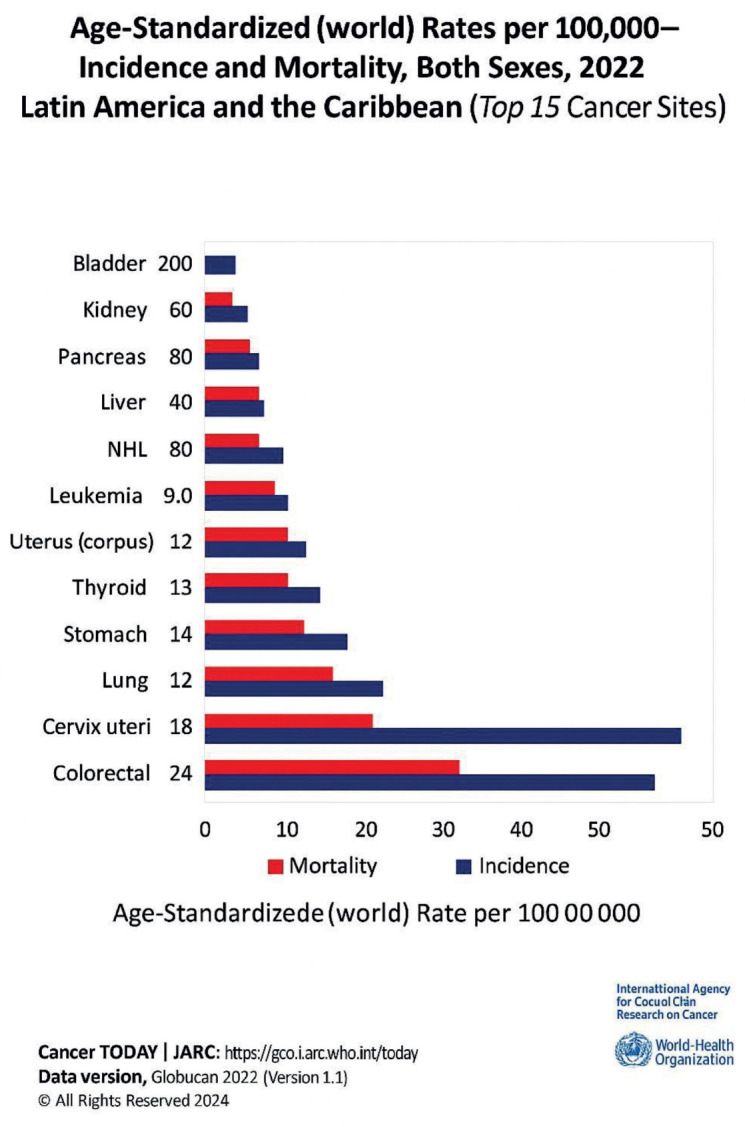
Main cancer localisation sites. It can be seen that breast cancer in women and prostate cancer in men have the highest morbidity and mortality rates.

Despite advances in diagnosis and treatment, CVDs remain the leading cause of death in women worldwide. According to the World Heart Federation, CVDs, which include heart disease and stroke, are the most common NCDs worldwide, responsible for nearly 20.5 million deaths. Alarmingly, more than 75% occur in low- and middle-income countries [7]. CVD is responsible for 35% of women's deaths each year, exceeding the rate of breast cancer by more than 13 times, greater than all cancers combined [7] (Figure 2).

It is known that breast cancer and CVD share risk factors, such as: age, diet, family history, alcohol consumption, hormone replacement, obesity/overweight, physical inactivity and tobacco use [8, 9]. Although breast cancer is not a reproductive milestone per se, treatment often alters reproductive function and compromises ovarian hormone production.

Cardio-oncology has emerged as clinical awareness of the broad cardiovascular implications of cancer and its treatments has grown. In a cohort from the *Surveillance, Epidemiology and End Results* cancer registry that included women with definitive treatment for localised breast cancer and who were alive 5 years after their initial diagnosis, the cumulative incidence of non-breast cancer mortality was almost seven times greater than the cumulative incidence of breast cancer mortality. CVD was the most common cause, affecting 30% of women [9].

Currently, there is an exponential growth of CVD in cancer patients, related to their longer survival and the use of oncological therapies that produce cardiovascular toxicity, which further increases the risk of CVD. This has prompted a multidisciplinary and novel approach in the field of cardio-oncology. The goal is to reduce CVD morbi-mortality resulting from the cardiotoxicity of oncology treatment. To achieve this, oncology patients' cardiovascular health is assessed comprehensively before starting treatment and their potential cardiotoxicity is monitored during and after oncology therapy. In addition, the aim is to ensure that the patient receives the first-line treatment for his or her disease. In this context, terms such as ‘preventive cardio-oncology’ and ‘permissive cardiotoxicity’ have been coined [10, 11].

In addition to the traditional link between the adverse effects of cancer therapies and cardiovascular health, the concept of ‘reverse cardio-oncology’ is emerging. This burgeoning field shifts the perspective by examining how CVD may influence the onset and progression of cancer. An increased likelihood of developing cancer has been observed in patients with pre-existing cardiovascular conditions, attributed to shared risk factors such as obesity, sedentary lifestyle and smoking. Underlying mechanisms such as chronic inflammation and clonal hematopoies is shed further light on the connections [7].

LAC are no strangers to the situation described above; however, the approach to the problem is at different stages. Multidisciplinary cardio-oncology groups have been established in some countries, but the current number is still insufficient and there is no regional approach [12]. This publication describes the epidemiological situation of cancer, as well as the state of development of cardio-oncology in the region.

## Epidemiology in LAC

The leading causes of death in the region in 2019 did not differ significantly from those in high-income regions, according to data from the World Health Organisation (WHO). CVD and cancer predominated, followed by stroke, Alzheimer's and other dementias, as well as chronic obstructive pulmonary disease [13].

In 2022, life expectancy in LAC, according to the Statista Research Department (2023), was 77 years for women and 70 years for men. In addition, South America had the highest sub-regional life expectancy, reaching 78 years for women and 71 years for men [14].

In 2019, the age-standardised mortality rate for cancer was estimated at 115.7 deaths per 100,000 population (excluding non-melanoma skin tumours) (Figure 2). This rate varied from country to country from 155.2 deaths per 100,000 population in Grenada to 68.7 deaths per 100,000 population in El Salvador. In most countries, age-standardised mortality rates for cancers are higher in men than in women, except in Bolivia, Guyana and El Salvador. Four types of malignant tumours appear in the list of the 15 leading causes of death: lung, colon and rectum, breast and prostate cancer [15].

Oncologic diseases in 2019 were responsible for 31.0 million years of life lost due to premature death (YLL), equivalent to 3,072 years per 100,000 population. The number of DALYs increased from 25.2 million years in 2000 to 31.0 million years in 2019. The top five cancer types that impacted DALYs were lung, breast, colon and rectal cancer, leukemia, lymphomas and multiple myeloma [16].

On the other hand, the LAC region has made significant progress in macroeconomic resilience over the last three decades. According to the World Bank report, the regional Gross Domestic Product (GDP) grew by 2.0% in 2023, slightly above the 1.4% previously projected [15]. However, these macroeconomic achievements did not translate into an increase in medical research in the region. Currently, only 0.65% of GDP is allocated to medical research and the region participates in only 5% of global cancer therapy clinical trials [13].

## Current status of cardio-oncology in LAC

A narrative summary was made of the epidemiology of cancer in the region during the last 5 years. Only three countries have national cancer incidence and mortality registries: Uruguay, Cuba and Costa Rica. Therefore, WHO and Pan American Health Organisation (PAHO) registries were used.

LAC show varying degrees of development of cardio-oncology, despite the support provided by the Ibero-Latin American Society of Cardio-oncology (iLACO) and the International Society of Cardio-oncology (IC-OS). This development has been limited to isolated groups in academic centers. However, Brazil and Argentina have made greater progress and have published national guidelines on the subject [17–20].

Professionals recognise the usefulness of multimodality cardiac diagnostic imaging in the evaluation of oncology patients before, during and after specific treatment for early detection of cardiotoxicity [21].

**No hay ninguna fuente en el documento actual.**). It is important to review the possibilities of radiological and nuclear techniques, considering the variability in the availability of the recommended technology between countries and even between different geographical areas of the same country [22].

European cardio-oncology guidelines recommend the use of echocardiography, especially 3D and *longitudinal Strain Global* to assess these patients. Cardiac magnetic resonance, is the second option, although its access is limited due to its high cost and lack of trained personnel. Isotopic ventriculography (ERNA) is an indication II-C, although it is a valid and necessary alternative in cases of poor ultrasound window due to its high reproducibility [21], but it is little used except in Panama, where its use predominates, and in El Salvador and Cuba in selected cases. In other countries, a low volume of requests for ANRT has been reported. Although atherogenesis is described among the cardiotoxic side effects of radiotherapy [23], where the role of myocardial perfusion (PM SPECT) is very useful in the early detection of ischemic coronary artery disease, its use in this type of patient is scarce.

Although regional cancer registries are considered for national statistics by sources such as Globocan and organisations such as PAHO, not all countries have national cancer registries due to high logistical costs. In addition, these registries are usually general statistical data, so they do not collect detailed information on diagnosis, treatment or toxicities [24]. At this point, we would like to highlight the Obelisco registry,

carried out by the Cardio-oncology Council of the Argentine Society of Cardiology, the first registry we know of on cardiotoxicity in Latin America [25]. However, to address this limitation, the Global Cardio-Oncology Registry, a prospective, multicenter, multinational registry that collects information on cardiotoxicities due to oncospecific treatments, has been created [26].

As part of the International Atomic Energy Agency (IAEA) technical cooperation project for LAC, *RLA6093: Strengthening regional capacities for the use of nuclear medicine techniques in a multimodal cardio-oncology approach in cancer patients (ARCAL CXCIII)*, a survey was carried out among the countries participating in the project to evaluate the current state of cardio-oncology in each country, considering a description of the situation during the first semester of 2024. Seventeen countries responded: Argentina, Brazil, Bolivia, Colombia, Chile, Costa Rica, Cuba, Dominican Republic, El Salvador, Guatemala, Honduras, Mexico, Nicaragua, Panama, Paraguay, Peru and Uruguay.

### Survey analysis

**Figure 3.**
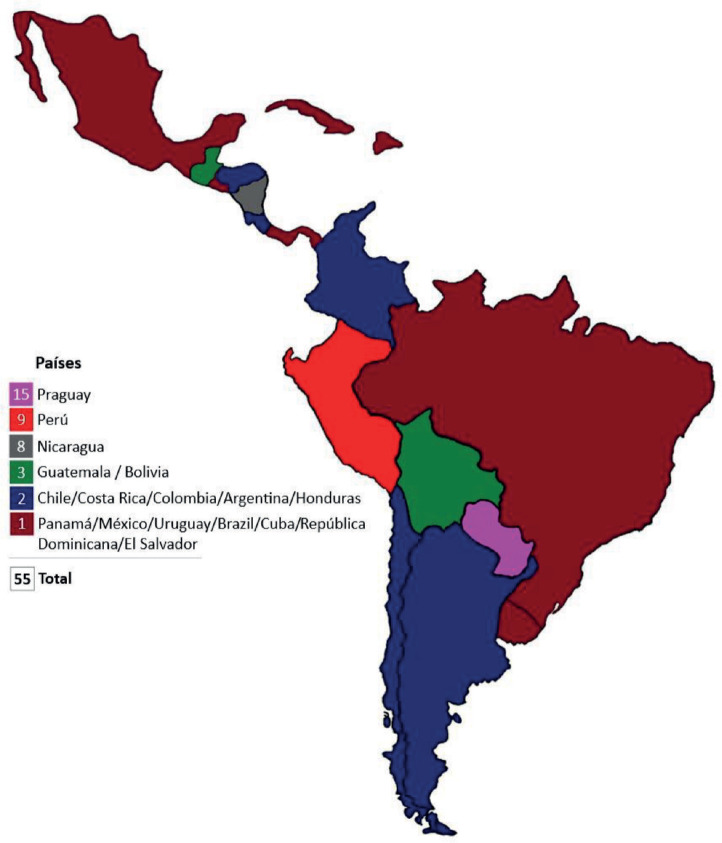
Number of respondents by countries participating in the survey.

The survey was answered by 55 professionals from the 17 countries included (Figure 3). Eighty-five percent of the participants were oncologists and cardiologists, while 11% were specialists in internal medicine. Most of these specialists work in general public hospitals (63.6%).

Only 34% report having a cardio-oncology service or group, which is explained by the lack of training of professionals in this field, as well as insufficient human and material resources and infrastructure.

Comprehensive cardio-oncologic evaluation before, during and after oncospecific treatment is reported in 16% of the respondents. The rest indicated that it is not performed frequently or only occasionally, which prevents us from obtaining a global stratification of cardiovascular risk and establishing measures for adequate primary prevention.

Patients are generally referred to cardiology when their treating physician (oncologist or hematologist) detects the presence of cardiovascular risk factors (40%), for a history of coronary artery disease (23.6%), as part of an institutional protocol or according to the planned therapeutic scheme (32%). The majority of respondents use the international guidelines of the related scientific societies, the most widely used being those of the American Society (ASCO) + European Society of Oncology (ESMO) in 27.2%. European cardio-oncology guidelines are used exclusively by 16.3%.

A high prevalence of breast, prostate, colon, lung and leukemia cancers was identified, coinciding with the statistics published by WHO. The usual treatment is surgery, chemotherapy and radiotherapy [27].

Eighty-four percent of respondents report the use of biomarkers for the detection of cardiotoxicity, the most commonly used being troponin and brain natriuretic peptide.

Echocardiography is the most widely used method to assess ventricular function (93%) and practically all consider it to be the fastest access diagnostic method. The most commonly used protocol for cardiotoxicity assessment is the 2D mode (47.2%). There was a low use of the global longitudinal *strain* measurement. Echocardiogram prior to potentially cardiotoxic treatment is performed in 31% of cases, only when there is any history in 15% of cases, while it is not performed in 2% of cases. The follow-up echocardiogram was performed depending on the scheme in 58% and according to evolution in 29%.

Isotopic ventriculography studies (ERNA) are used in case of doubts or discrepancies; it is considered among the options in 45.4%, while 5.4% report it as the only option. Myocardial perfusion studies (PM SPECT) are only performed when coronary artery disease is suspected. Myocardial ischemia is detected in approximately 15% of these patients.

Cardioprotection is indicated in most patients (65.5%) with one of the existing schemes.

The use of liposomal anthracyclines is not widely used due to their high cost and/or lack of coverage in the health system.

A high percentage (65.5%) consider that training for evaluation and detection of cardiotoxicity is insufficient or absent. Only one professional considers that he/she has expert training. There is little access to formal education in cardio-oncology in 62% and no access at all in 22%. Only 14.5% reported being very accessible.

## Recommendations

## Conclusion

Improvements in cancer treatments, increased survival rates and the potential for cardiac side effects of these treatments have led to greater collaboration between: oncology, cardiologists and hematologists, as well as the development of cardio-oncology clinics. This collaboration is important because these specialists ensure greater patient satisfaction, assist physician teams in making complex treatment decisions and diagnose cardiac complications early.

Cardio-oncology in LAC works in isolated groups and with diverse developments. We hope that, in the coming years, thanks to compliance with these recommendations and the support of organisations such as the IAEA, we will achieve uniform development throughout the region and the incorporation of the different cardio-oncology societies and working groups into international societies such as SIAC, iLACO and IC-OS, which will allow us to position ourselves in this increasingly important and necessary area, both in our countries and in the region, to promote the development of this new subspecialty.

## Conflicts of interest

We have no conflicts of interest.

## Funding

No funding has been received for the preparation of the manuscript.

## Figures and Tables

**Table.** Recommendations

1. Continuing education: It is important to organise continuing education projects for physicians, nurses, graduates, psychologists and other health professionals related to cardio-oncology. These projects can include *webinars*, *e-learning* courses and face-to-face events. 2. Local cardio-oncology groups: Countries should encourage the creation of local cardio-oncology groups. Ideally, these groups should be composed of cardiologists, oncologists, hematologists, nurses and psychologists. 3. Social networks for cardio-oncology topics: Groups can be organised on social networks, such as WhatsApp, to discuss topics related to cardio-oncology. The support of the IAEA communications group can be valuable in this regard. 4. Inclusion in training programs: It is essential to include the subject of cardiotoxicity in oncology, hematology and cardiology training programs. This ensures that professionals are trained to address these issues. 5. Consensus for Latin America: A specific consensus should be established for Latin America, based on international guidelines but adapted to the situation in the region. This will help to unify the concepts related to the initial cardiologic evaluation and follow-up of cardio-oncology patients. 6. Latin American Research Network: It would be beneficial to create a Latin American network that encourages clinical analysis and publication of research on the characteristics of our population in the context of cardio-oncology.
